# Obscure Gastrointestinal Bleeding in an Adolescent Masked by Gastric Erosions: Meckel’s Diverticulitis Complicated by Adhesive Obstruction and Small Bowel Perforation

**DOI:** 10.7759/cureus.100255

**Published:** 2025-12-28

**Authors:** Henny R Thanki, Suril Vithalani

**Affiliations:** 1 Department of Colorectal Surgery, Gujarat Medical Education and Research Society (GMERS) Medical College and Hospital, Junagadh, IND; 2 Department of Colorectal Surgery, Zydus Hospitals, Ahmedabad, IND

**Keywords:** adhesive small bowel obstruction, adolescent surgery, diagnostic challenges, diagnostic delay, ectopic gastric mucosa, gastric erosion, gastrointestinal hemorrhage / etiology, meckel’s diverticulum, obscure gastrointestinal bleeding, small bowel perforation

## Abstract

Meckel’s diverticulum is the most frequent congenital anomaly of the gastrointestinal tract, yet its identification can be challenging when symptoms mimic those of more common conditions. In adolescents, this often leads to misdirection during the diagnosis and delays in identifying the true source of bleeding. A 16-year-old male presented with recurrent melena and abdominal pain. Initial esophagogastroduodenoscopy (EGD) revealed mild gastric erosions, and colonoscopy was normal, prompting treatment for gastritis and temporary symptom resolution. Ten months later, he returned with massive gastrointestinal bleeding. CT imaging suggested Meckel's diverticulitis, which was confirmed intraoperatively, and the patient underwent segmental small bowel resection with appendectomy. Histopathology confirmed Meckel's diverticulitis, which contained ectopic gastric mucosa. This case highlights the diagnostic pitfalls of obscure gastrointestinal bleeding in young patients. Mild gastric erosions should not preclude the evaluation of the small bowel when bleeding persists. Early consideration of Meckel’s diverticulum can help prevent avoidable morbidity and ensure timely, effective management.

## Introduction

Meckel’s diverticulum is the most frequently encountered congenital abnormality of the gastrointestinal tract and is present in roughly 2% of the population [1]. Although many individuals remain asymptomatic, a range of complications can arise, including ulceration, bleeding, intussusception, obstruction, perforation, and, less commonly, vesicodiverticular fistulae or neoplasms. While bleeding related to ectopic gastric mucosa is well recognized in younger children, identifying this condition in adolescents is often more challenging because their symptoms frequently mimic more common conditions such as gastritis or inflammatory bowel disease [2]. This symptomatic overlap often leads to diagnostic misdirection, as occurred in this case, where initial findings of mild gastric erosions diverted attention away from the small bowel. Adolescents often exhibit symptoms that do not clearly align with typical pediatric or adult presentations, further contributing to diagnostic uncertainty and delayed recognition. In this context, the mild gastric erosions observed on esophagogastroduodenoscopy (EGD) may create a misleading sense of reassurance, discouraging clinicians from further evaluating the small bowel and delaying identification of the true pathology. Recognizing these pitfalls is important, as delayed diagnosis may increase the risk of recurrent bleeding or additional complications. Understanding how misleading endoscopic findings can influence clinical reasoning underscores the educational value of this case.

## Case presentation

**Figure 1:**
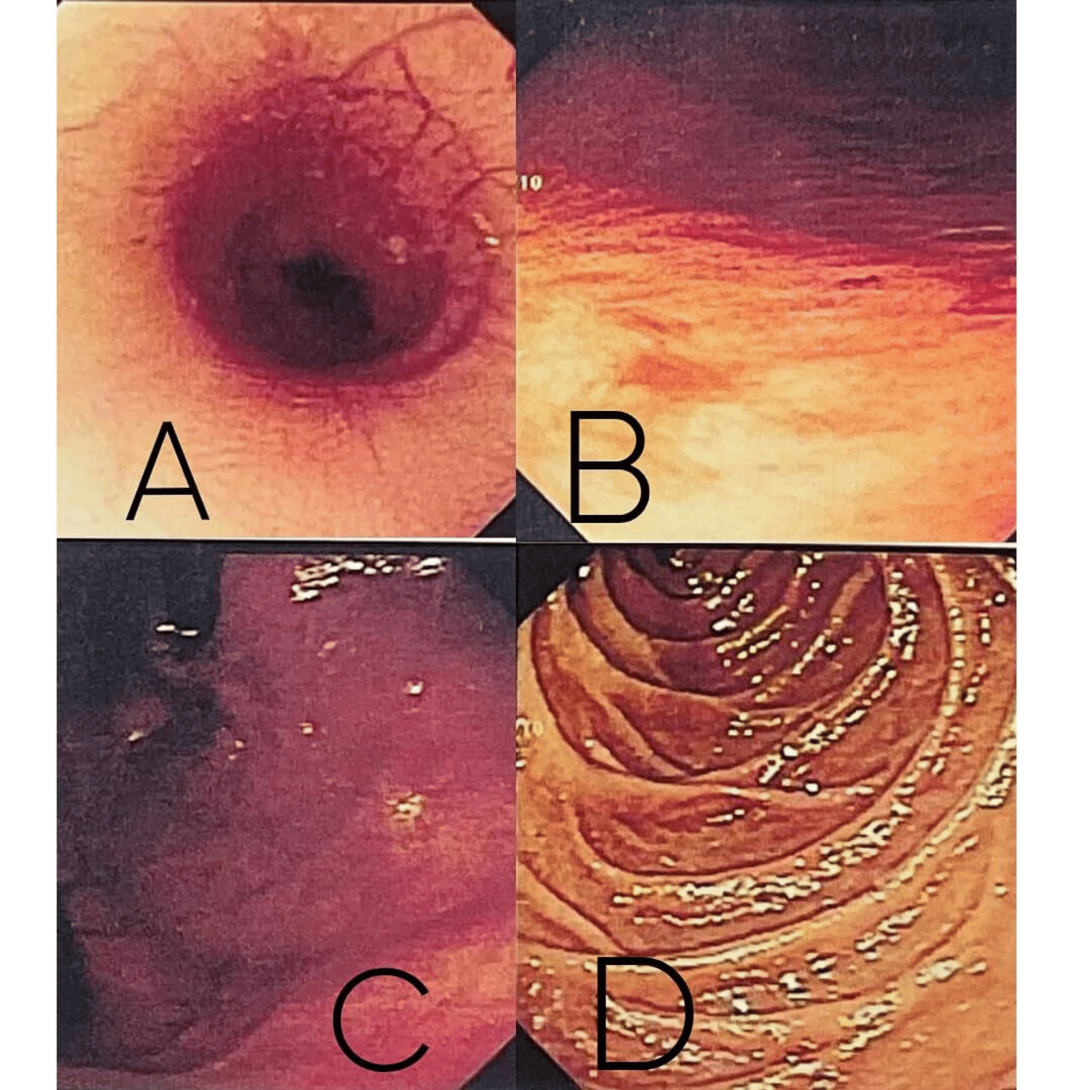
Esophagogastroduodenoscopy (EGD) findings at initial presentation. A: distal esophagus and gastroesophageal junction showing normal mucosa with no ulceration or inflammation; B: gastric body demonstrating mild superficial erosions without active bleeding; C: antrum/prepyloric region of the stomach with erythematous mucosa and superficial erosions; D: second portion of the duodenum (D2) showing normal mucosal folds and no bleeding source.

A 16-year-old male initially presented with recurrent episodes of melena and abdominal pain lasting four to five days, accompanied by headache, backache, dizziness, and rhinitis. An initial EGD revealed erosions in the body of the stomach, while a colonoscopy was normal. Consequently, the patient was diagnosed with gastritis and discharged; symptoms gradually resolved after one month of conservative management. Figures 1A-1D illustrate the EGD findings, which showed mild gastric erosions without any active bleeding.

**Figure 2:**
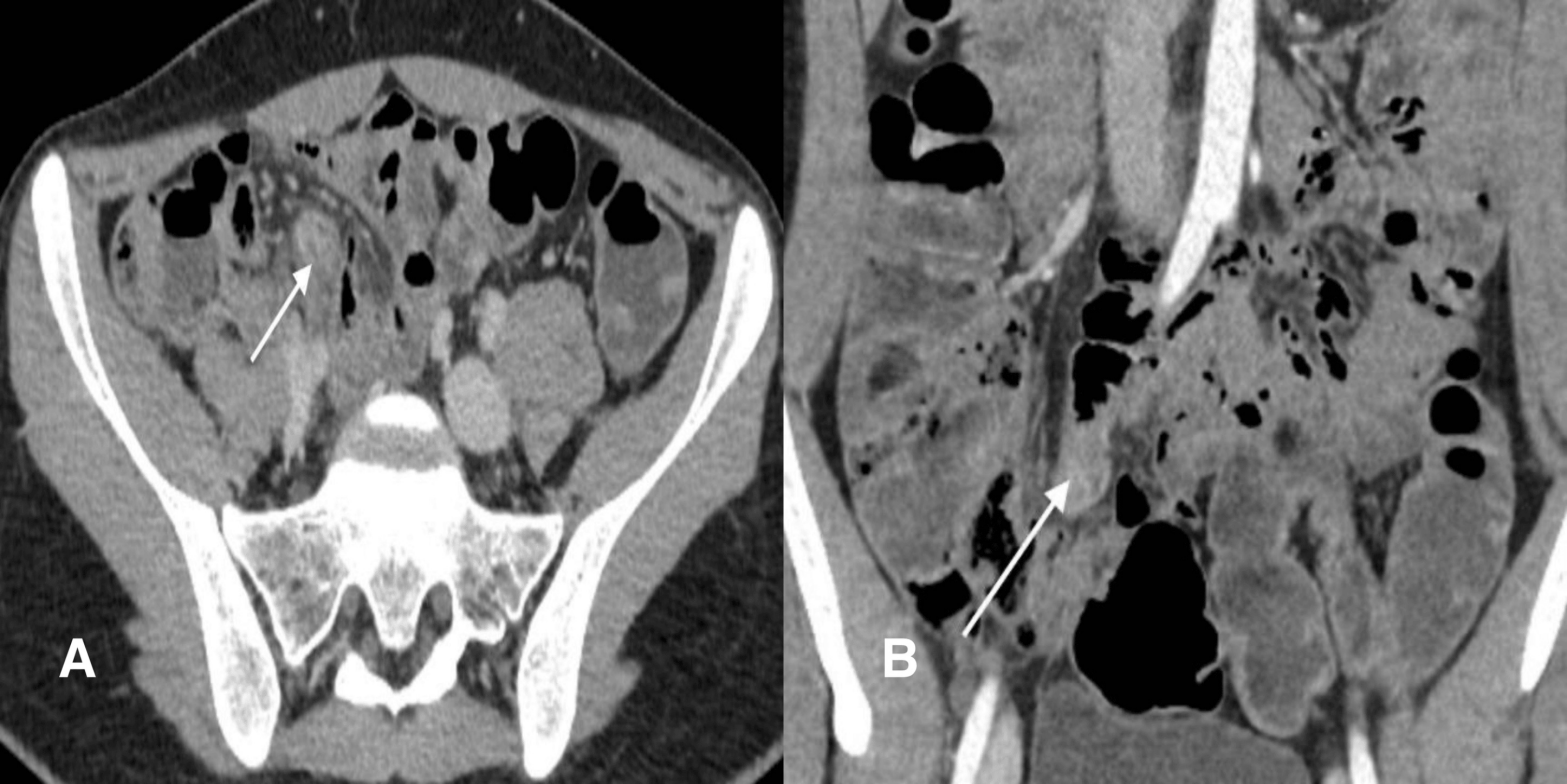
Contrast-enhanced CT abdomen. A: axial CT image showing a blind-ending tubular structure arising from the distal ileum (arrow) with surrounding inflammatory fat stranding, suggestive of Meckel's diverticulitis; B: coronal CT image demonstrating the same blind-ending ileal outpouching (arrow), with adjacent inflammatory changes, supporting the diagnosis of Meckel's diverticulitis.

Ten months later, he experienced a recurrence of massive gastrointestinal bleeding with three to four episodes of melena per day. Contrast-enhanced computed tomography (CECT) of the abdomen and pelvis revealed a blind-ended tubular structure arising from the ileum, indicating Meckel's diverticulitis. A preoperative diagnosis was suspected radiologically; however, definitive confirmation was achieved intraoperatively during diagnostic laparoscopy. As shown in Figure 2, the CECT scan identified a blind-ended tubular outpouching from the ileum, suggestive of Meckel's diverticulum.

**Figure 3:**
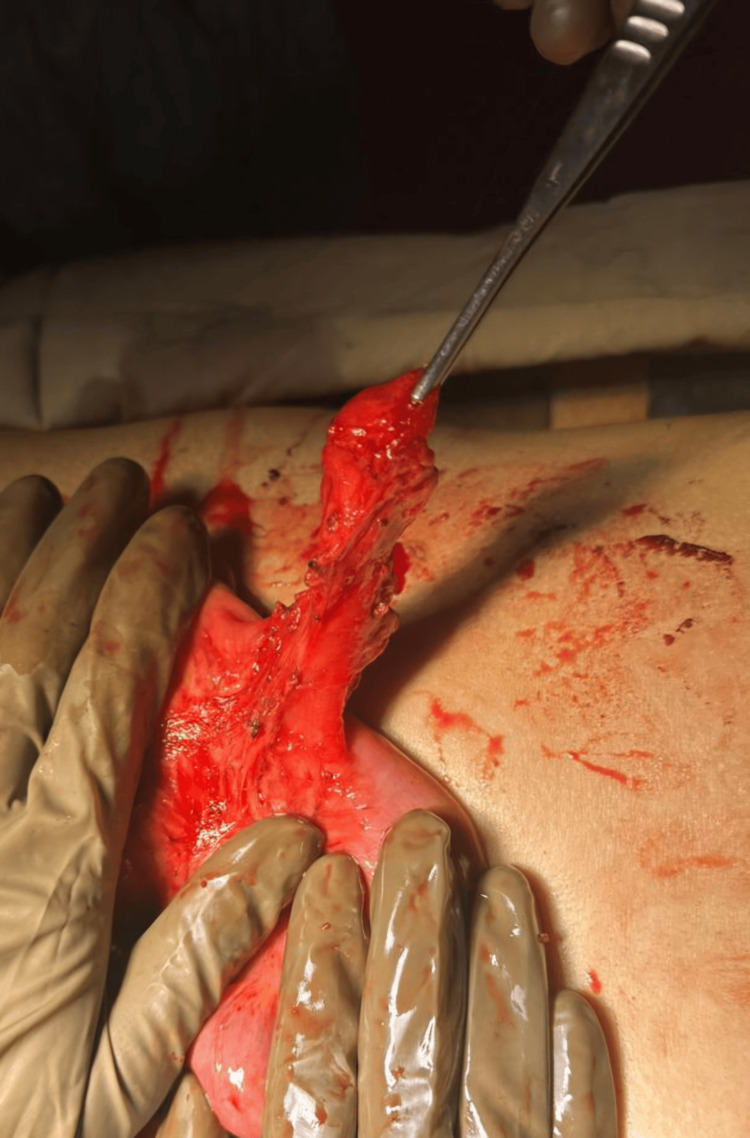
Intraoperative image showing an inflamed Meckel's diverticulum before resection.

Diagnostic laparoscopy revealed an inflamed Meckel's diverticulum with an inflamed appendix. Although the procedure was initiated laparoscopically, dense inflammatory adhesions around the Meckel’s diverticulum and its proximity to the inflamed appendix limited safe mobilization. Therefore, the surgery was converted to an open laparotomy to allow better exposure, and a segmental small bowel resection with side-to-side anastomosis and a concomitant appendectomy was performed. Figure 3 depicts the intraoperative appearance of the inflamed Meckel's diverticulum before resection.

Histopathological examination confirmed the presence of Meckel's diverticulitis containing ectopic gastric mucosa and concurrent chronic appendicitis. Postoperatively, the patient was managed with nasogastric decompression, intravenous fluids, bowel rest, and a stepwise reintroduction of diet alongside standard antibiotic and analgesic therapy.

**Figure 4:**
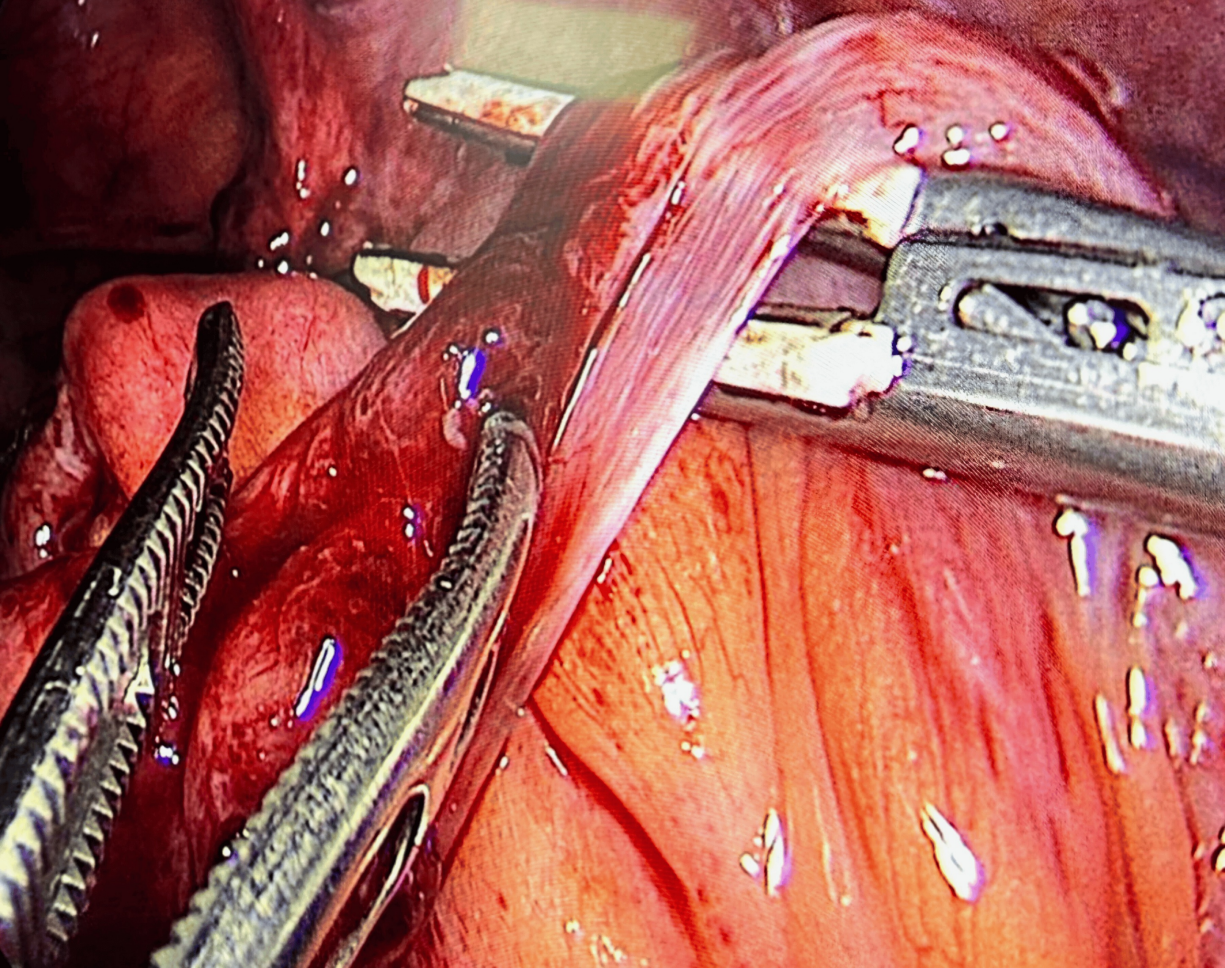
Laparoscopic view showing an adhesive band causing small bowel obstruction.

Ten days postoperatively, the patient was readmitted with complaints of recurrent bilious vomiting. CT imaging suggested subacute small bowel obstruction, which resolved over four days of conservative management. Six months later, he presented with acute small bowel obstruction secondary to an adhesive band, complicated by a small bowel perforation. Emergency laparoscopic adhesiolysis was initiated, and the adhesive band was successfully divided laparoscopically. However, the presence of a small bowel perforation necessitated conversion to open laparotomy to allow safe inspection of the bowel and primary repair. Figure 4 demonstrates the adhesive band responsible for the patient's small bowel obstruction.

Postoperative analgesia included regional (spinal) pain control delivered via continuous infusion during the early recovery period, along with nasogastric decompression, intravenous hydration, and gradual advancement of diet. The patient made a full recovery after this second surgery.

## Discussion

Meckel's diverticulum is the most common congenital anomaly of the gastrointestinal tract due to persistence of the proximal part of the omphalomesenteric duct [1]. Although present in about 2% of the population, the majority remain asymptomatic, and only a small proportion develop clinical manifestations [2]. The symptomatic presentation varies and may include bleeding, inflammation, obstruction, or, rarely, perforation [3]. Bleeding is a particularly characteristic presentation in the pediatric population, whereas symptomatic presentation in adolescents and adults is relatively uncommon, making timely diagnosis more difficult in these age groups [4,5]. Because the clinical features of Meckel's diverticulum often mimic more prevalent gastrointestinal conditions, diagnosing it demands a high index of suspicion [6].

In this case, bleeding was explained by the ectopic gastric mucosa present within the diverticulum: acid produced there can erode nearby ileal mucosa and cause significant bleeding [3,7]. Standard endoscopic evaluation by EGD and colonoscopy often fails to identify the source of bleeding because the distal ileum is beyond their visual reach [8]. In some cases, incidental or minor findings such as mild gastric erosions may lead to a misleading diagnosis, providing false assurance that the bleeding source has been identified [9]. Imaging techniques, such as a CT, can be useful when diverticulitis or obstruction is present. CT may suggest diverticulitis when inflammation is present, but its sensitivity can be variable in non-inflamed or complicated cases [7]. Technetium-99m pertechnetate scan is sensitive for detecting ectopic gastric mucosa but is less reliable in complicated cases such as inflammation or perforation. Therefore, when endoscopic or imaging studies are inconclusive, diagnostic laparoscopy becomes an essential tool for identifying the underlying pathology.

Massive hemorrhage due to Meckel's diverticulum is a well-documented presentation in young children but remains distinctly uncommon in adolescents. As a result, when teenagers present with melena, clinicians often prioritize more common diagnoses such as peptic ulcer disease or erosive gastritis, as occurred in this case [2,10]. This highlights how minor gastric abnormalities can misdirect clinical reasoning, especially when symptoms temporarily improve after empirical therapy. Additionally, CT imaging does not always clearly distinguish Meckel's diverticulum from other inflammatory conditions involving the ileum, particularly in early or subtle presentations [5]. Therefore, recurrent or persistent bleeding despite benign EGD and colonoscopy findings should prompt clinicians to broaden their differential diagnosis and include Meckel's diverticulum.

While resection treats the diverticulum, the patient’s later adhesive small-bowel obstruction underscores that even uncomplicated resections can lead to adhesive disease, a common cause of later readmission and morbidity [11]. Although early partial obstruction may resolve conservatively, progression to perforation, as in this case, is uncommon but clinically significant. This progression highlights the unpredictable nature of postoperative adhesive disease, even after minimally invasive procedures [12].

This case provides several important clinical lessons. First, adolescents presenting with recurrent, unexplained gastrointestinal bleeding should be evaluated for Meckel's diverticulum, particularly when routine endoscopy fails to identify a bleeding source. Second, seemingly insignificant mucosal abnormalities, such as gastric erosions, should not prematurely exclude distal small bowel pathology. Third, patients undergoing small bowel surgery require continuous long-term observation, as postoperative adhesion complications may occur months or years after the procedure. Overall, this case reinforces the need for vigilance when evaluating obscure bleeding in adolescents and the value of maintaining a broad differential diagnosis to prevent avoidable morbidity.

## Conclusions

This case highlights the diagnostic pitfall associated with obscure gastrointestinal bleeding in young patients, particularly when misleading findings such as mild gastric erosions are present. It emphasizes that benign or incomplete endoscopic findings should not delay evaluation of the small bowel when bleeding persists or recurs. The case also demonstrates the significant morbidity linked to delayed recognition of Meckel’s diverticulum, including severe hemorrhage and later adhesive complications. In addition to ensuring timely diagnosis, clinicians must remain aware of the potential for delayed postoperative adhesion-related obstruction or perforation, even after successful surgery.
